# Light-sheet autofluorescence lifetime imaging with a single-photon avalanche diode array

**DOI:** 10.1117/1.JBO.28.6.066502

**Published:** 2023-06-21

**Authors:** Kayvan Samimi, Danielle E. Desa, Wei Lin, Kurt Weiss, Joe Li, Jan Huisken, Veronika Miskolci, Anna Huttenlocher, Jenu V. Chacko, Andreas Velten, Jeremy D. Rogers, Kevin W. Eliceiri, Melissa C. Skala

**Affiliations:** a Morgridge Institute for Research, Madison, Wisconsin, United States; b University of Wisconsin, Department of Electrical and Computer Engineering, Madison, Wisconsin, United States; c University of Wisconsin, Department of Biochemistry, Madison, Wisconsin, United States; d Georg-August-University Göttingen, Department of Biology and Psychology, Göttingen, Germany; e University of Wisconsin, Department of Medical Microbiology and Immunology, Madison, Wisconsin, United States; f Rutgers New Jersey Medical School, Center for Cell Signaling, Newark, New Jersey, United States; g Rutgers New Jersey Medical School, Department of Microbiology, Biochemistry and Molecular Genetics, Newark, New Jersey, United States; h University of Wisconsin, Department of Pediatrics, Madison, Wisconsin, United States; i University of Wisconsin, Laboratory for Optical and Computational Instrumentation, Madison, Wisconsin, United States; j University of Wisconsin, Department of Biostatistics and Medical Informatics, Madison, Wisconsin, United States; k University of Wisconsin, McPherson Eye Research Institute, Madison, Wisconsin, United States; l University of Wisconsin, Department of Ophthalmology and Visual Sciences, Madison, Wisconsin, United States; m University of Wisconsin, Department of Biomedical Engineering, Madison, Wisconsin, United States

**Keywords:** autofluorescence, single-photon avalanche diode, fluorescence lifetime imaging microscopy, light-sheet microscopy, nicotinamide adenine dinucleotide (phosphate)

## Abstract

**Significance:**

Fluorescence lifetime imaging microscopy (FLIM) of the metabolic co-enzyme nicotinamide adenine dinucleotide (phosphate) [NAD(P)H] is a popular method to monitor single-cell metabolism within unperturbed, living 3D systems. However, FLIM of NAD(P)H has not been performed in a light-sheet geometry, which is advantageous for rapid imaging of cells within live 3D samples.

**Aim:**

We aim to design, validate, and demonstrate a proof-of-concept light-sheet system for NAD(P)H FLIM.

**Approach:**

A single-photon avalanche diode camera was integrated into a light-sheet microscope to achieve optical sectioning and limit out-of-focus contributions for NAD(P)H FLIM of single cells.

**Results:**

An NAD(P)H light-sheet FLIM system was built and validated with fluorescence lifetime standards and with time-course imaging of metabolic perturbations in pancreas cancer cells with 10 s integration times. NAD(P)H light-sheet FLIM *in vivo* was demonstrated with live neutrophil imaging in a larval zebrafish tail wound also with 10 s integration times. Finally, the theoretical and practical imaging speeds for NAD(P)H FLIM were compared across laser scanning and light-sheet geometries, indicating a 30× to 6× acquisition speed advantage for the light sheet compared to the laser scanning geometry.

**Conclusions:**

FLIM of NAD(P)H is feasible in a light-sheet geometry and is attractive for 3D live cell imaging applications, such as monitoring immune cell metabolism and migration within an organism.

## 1 Introduction

Cell migration and single cell metabolism are popular areas of investigation across immunology, cancer research, and developmental biology.^1^ However, a significant bottleneck exists in 3D imaging of metabolic features in moving cells throughout a live model organism, such as the zebrafish. Current methods require sample destruction (e.g., mass spectrometry, flow cytometry, and histology) that removes 3D context and prevents time-course studies that follow the fate of the same cell, or fluorescent reporters that require sample manipulation.^2^ Additionally, fluorescent reporters often provide a binary on/off indicator of expression rather than a continuous variable of dynamic cell state, even though most cells are known to function on a spectrum of activity.

The reduced form of nicotinamide adenine dinucleotide (phosphate) [NAD(P)H] is a naturally fluorescent metabolic co-factor involved in hundreds of reactions within the cell (NADH and NADPH are optically indistinguishable and their fluorescence is collectively referred to as NAD(P)H).^2^^–^^13^ The fluorescence lifetime of NAD(P)H is distinct in the free (short lifetime) and protein-bound (long lifetime) conformations, so fluorescence lifetime imaging microscopy (FLIM) of NAD(P)H provides information on protein-binding activities, preferred protein-binding partners, and other environmental factors, such as pH and oxygen, at a single cell level.^2^^,^^10^^,^^12^^,^^14^^,^^15^ FLIM of NAD(P)H is advantageous for imaging samples that are difficult to label with fluorescent reporters, for *in vivo* imaging of preclinical models, and for visualizing fast biological processes. For example, NAD(P)H FLIM can probe the temporal and spatial regulation of macrophage metabolism during tissue damage and repair in live zebrafish.^11^ NAD(P)H FLIM typically relies on two-photon (2P) laser scanning microscopy, which provides intrinsic optical sectioning, improved penetration depth, and high spatial resolution with minimal phototoxicity.^16^^,^^17^ However, autofluorescence FLIM traditionally suffers from long acquisition times and limited spatial views, partly due to the low fluorescence quantum yield ($\Phi_{f}$ for NAD(P)H is between 0.02 and 0.10 depending on protein binding) as well as lower molar absorption coefficient [ϵ for NAD(P)H is $6200\;{\mathrm{cm}}^{-1}\;M^{-1}$], which makes the autofluorescence signal hundreds of times dimmer than that of conventional fluorophores (e.g., the green fluorescent protein [GFP] has $\Phi_{f}=0.79$ and $\epsilon =25500\;{\mathrm{cm}}^{-1}\;M^{-1}$).^18^^–^^20^ There is a significant opportunity to develop new NAD(P)H FLIM imaging hardware to better understand 3D cellular dynamics *in vitro* and *in vivo*.

Single-photon avalanche diodes (SPAD) are solid-state photodetectors with high photon counting and time-resolving capabilities.^21^ SPADs are semiconductor p–n junctions reverse-biased above their breakdown voltage such that a single-photon event can create an electron–hole pair and trigger a self-sustaining avalanche current. Advances in SPAD technology have led to the development of multipixel arrays with on-chip integrated timing electronics [such as time-to-digital converters (TDC)] capable of distinguishing single-photon arrival times.^21^ SPAD arrays have numerous applications in biophotonics, including fluorescence correlation spectroscopy,^22^^–^^24^ Raman spectroscopy,^25^^–^^27^ spectrally resolved FLIM,^28^ and optical tomography.^29^^–^^31^ Larger SPAD arrays (>32×32) have also been used extensively for FLIM in combination with multifocal multiphoton excitation, providing exceptional speed advantages while maintaining the spatial and temporal resolution of traditional single-beam scanning microscopes.^32^^–^^35^ Other application examples include real-time widefield FLIM with time-correlated single photon counting (TCSPC) or time-gated sensing to image standard fluorescence solutions and quantum dots,^36^^,^^37^ fluorescently labeled cells,^38^^,^^39^ and fungal spores.^40^ High-speed time domain FLIM with SPAD arrays has been used for rapid tumor phantom margin measurements^41^ and to distinguish labeled vasculature^42^ and melanomas in live mice.^43^ Other geometries and biophotonics applications are detailed in the extensive review by Bruschini et al.^21^

Although SPAD arrays can be easily integrated into widefield microscopes, these microscopes lack optical sectioning capabilities and therefore offer poor sensitivity for NAD(P)H FLIM, which targets a weak autofluorescence signal over a high background. Light-sheet microscopy, also known as selective plane illumination microscopy (SPIM), uses a light sheet perpendicular to the imaging axis to illuminate the focal plane and achieve optical sectioning.^44^ Light-sheet microscopy is an attractive alternative to widefield or laser scanning systems because it provides 3D optical sectioning along with fast volumetric imaging due to the following features: first, SPIM enables higher throughput due to parallel acquisition of image pixels; second, out-of-focus sample exposure and photobleaching are eliminated; finally, SPIM provides better excitation efficiency and lower irradiance compared to scanning a diffraction-limited spot in epi-illumination geometry, in turn lowering the light dose and phototoxicity relative to single-photon confocal systems.^44^^–^^46^ Light-sheet FLIM has been performed on GFP-labeled cancer cell spheroids and *C. elegans* using microchannel plate photomultiplier tube detectors^47^ and live transgenic zebrafish using a frequency-domain two-tap CMOS FLIM camera.^48^ Gated optical image intensifiers have been used in conjunction with CMOS^49^ or CCD^50^ cameras on SPIM-FLIM systems to image GFP-labeled live zebrafish^49^ or canine kidney cysts,^50^ respectively. To our knowledge, autofluorescent NAD(P)H FLIM has not yet been performed using light-sheet systems.

Here we demonstrate the first use of a commercially available SPAD array for detecting NAD(P)H autofluorescence in a light-sheet geometry. To improve image quality, the SPAD image from the FLIM arm of the light-sheet system was upscaled with the CMOS image from the intensity arm of the light-sheet system. The sensitivity of the NAD(P)H light-sheet FLIM system was confirmed with metabolic perturbations to pancreatic cancer cells with 10 s integration times, and the NAD(P)H light-sheet FLIM system was demonstrated *in vivo* with live neutrophil imaging in a wounded zebrafish tail, also at 10 s integration times. Finally, the theoretical and practical imaging speeds for NAD(P)H FLIM are compared across laser scanning and light-sheet geometries, indicating a 30× to 6× acquisition speed advantage for the light-sheet compared to the laser scanning geometry.

## 2 Methods

### 2.1 SPAD Array

A FLIMera SPAD array camera (Horiba Scientific) was used for NAD(P)H FLIM.^51^ The array consists of 192×128  pixels with dedicated TDC electronics per pixel for TCSPC. Each pixel TDC has a 12-bit quantized (4096 time bins) time axis with a temporal resolution (bin width) of 41.1 ps. The SPAD pixels have a pitch of 9.2  μm vertically and 18.4  μm horizontally (1.75  mm×2.35  mm sensor size), a fill factor of 13%, and median dark count rate (DCR) of 34 counts per second (cps). The instrument response function (IRF) of the device has a full-width at half-maximum (FWHM) of 380 ps. The FLIMera was controlled using Horiba EzTime Image software, with raw photon arrival time data streamed for 10 s detection periods to computer memory and subsequently saved to HDF5 files for post-processing. This photon stream saving mode provided complete raw data for custom analysis but constrained the effective multiframe acquisition speed by creating an avoidable time delay between successive frames (due to data being saved to disk in photon stream mode, requiring 32 bits per photon event, resulting in multigigabyte saved files as opposed to the equivalent 3D histogram, which would require a few megabytes). Conversely, integration times per image were constrained by the fill factor (13%) and photon detection probability (34%) of the sensor (i.e., detection efficiency).

### 2.2 Multidirectional Selective Plane Illumination Microscopy with SPAD Array

**Fig. 1:**
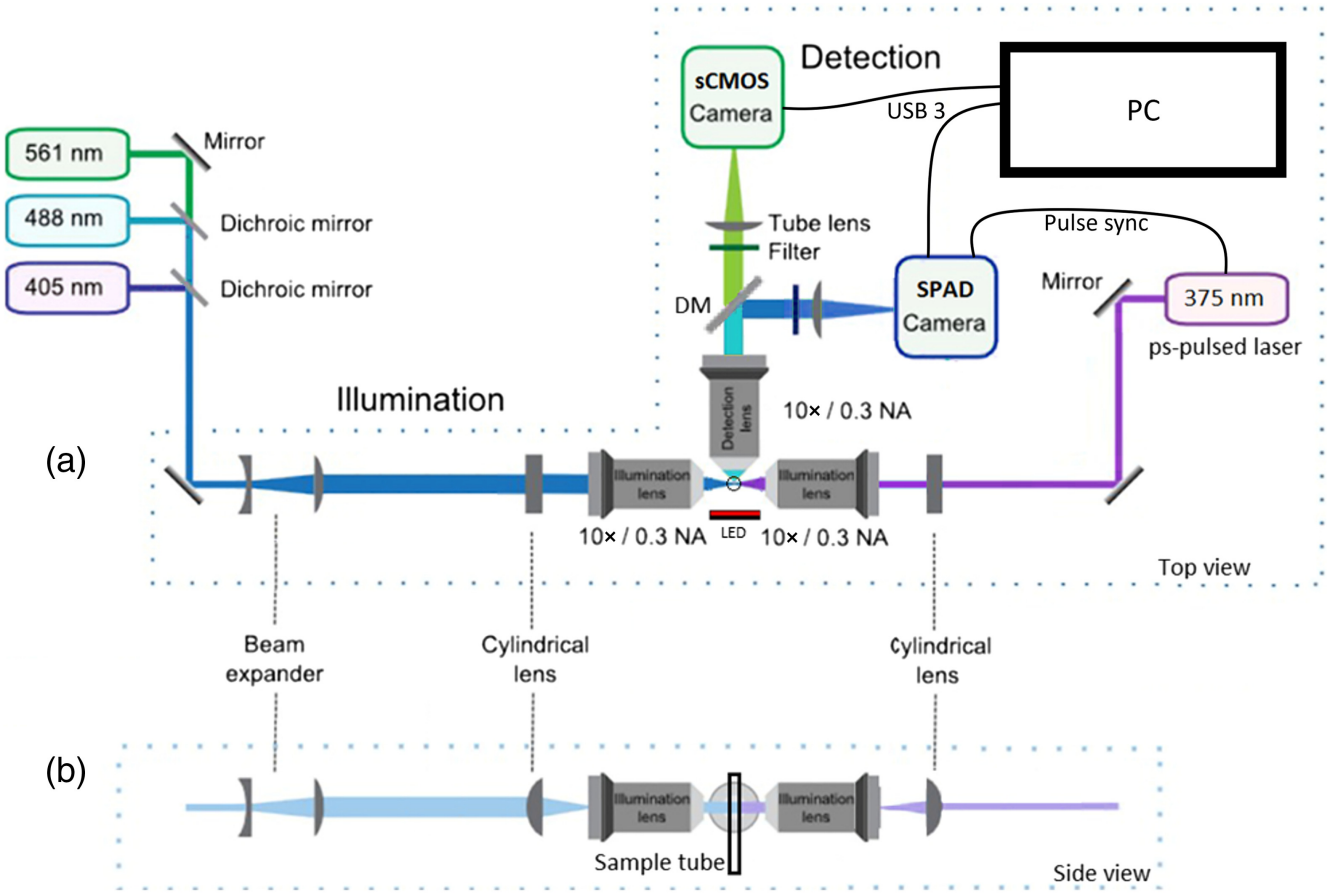
Schematic of the light-sheet FLIM microscope (adapted with permission from Ref. 52). (a) Top view of the SPIM system. The right illumination arm uses a 375 nm ps-pulsed diode laser operated at 50 MHz repetition rate to excite NAD(P)H fluorescence via a 200  μm wide light sheet, while the time-resolved SPAD camera images the NAD(P)H autofluorescence lifetime (after a 495 nm LP DM and a 440/80  nm filter) and the sCMOS camera images the NAD(P)H intensity (using a 530/55  nm filter). The left illumination arm uses a bank of CW lasers to excite other fluorophores via a 1.5 mm wide light sheet, while emissions are imaged on the sCMOS camera. A red LED provides trans-illumination for bright field imaging. (b) Side view of the beams in the illumination arms and the orientation of the sample tube. DM, dichroic mirror.

The mSPIM system is detailed in Fig. 1,^52^ with multidirectional capabilities maintained in the intensity arm only. Illumination was achieved using two opposing light sheets. Three 10×/0.3 NA water immersion objective lenses (CFI60, Nikon Instruments, Inc.) were used for illumination and detection. The “Flamingo” T-SPIM design^53^ was modified to incorporate a QuixX 375-70PS picosecond-pulsed diode laser (Omicron-Laserage Laserprodukte GmbH) in the right illumination arm for NAD(P)H excitation. The laser produced 90 ps pulses at a 50 MHz repetition rate with an average power of 0.4 mW at the sample. The output beam had a diameter of 1 mm which was used along with a f=100  mm cylindrical lens (Thorlabs) to underfill a 10×/0.3 NA objective lens, generating a 200  μm-wide light sheet with 9  μm waist thickness that matched the FLIMera sensor size (right arm, Fig. 1). A bank of continuous-wave lasers (TOPTICA) was used to generate a 1.5 mm-wide light sheet (left arm, Fig. 1) for targeting other fluorescent labels for intensity imaging using a larger sCMOS sensor (13.3  mm×13.3  mm) with 2048×2048  pixels, 6.5  μm pitch (Panda 4.2, PCO GmbH). Brightfield trans-illumination was achieved using a red LED and imaged using the sCMOS sensor.

NAD(P)H was excited at 375 nm (pulsed laser, 50 MHz, integration time = 10 s on SPAD array) and mCherry at 561 nm (CW laser, integration = 1 s on sCMOS camera). Emissions were split by a 495 nm long-pass dichroic mirror (DM); NAD(P)H was captured using a 440/80 nm bandpass filter on the SPAD array; and a lower NAD(P)H intensity signal was collected through a 530/55  nm bandpass filter on the sCMOS camera (integration time = 1 s). Fluorescence from mCherry and brightfield images was captured through a 650/60  nm bandpass filter on the sCMOS camera using illumination from the 561 nm CW laser light sheet or the red LED brightfield, respectively.

### 2.3 System Characterization and Calibration Measurements

The combined IRF of the pulsed laser source (Omicron QuixX 375-70PS) and the SPAD array camera (Horiba FLIMera) was measured in an epi-illuminated widefield microscope setup by imaging a retroreflector target. A fraction of the light from excitation laser pulses (with 50 MHz pulse repetition rate) was imaged onto the SPAD array. The non-zero tail of the recorded pixels IRF was used to estimate the DCR of individual pixels.

Standard fluorescent samples including a saturated solution of coumarin 6 (Sigma-Aldrich) in ethanol, Fluoresbrite^®^ Yellow-Green (YG), and Bright Blue (BB) microspheres (Polysciences) were mounted in fluorinated ethylene-propylene (FEP) tubes and imaged on the light-sheet FLIM system to test the accuracy of the FLIM measurements against reference values.

### 2.4 Data Processing

The HDF5 photon stream files were converted to 3D histograms of photon arrival times in MATLAB (MathWorks). The histograms were pre-processed to correct for the SPAD sensor artifacts, i.e., variable DCR and timing skew across the sensor pixels.^54^ The DCR was estimated for each pixel from the tail of the decay (i.e., the last 2 ns of the decay) as the offset variable for the lifetime fitting algorithm. The timing skew was corrected by applying a circular shift to each pixel decay histogram by a value (up to 2.5 ns) estimated from cross-correlation maximization of each pixel decay with a reference decay (measured from the coumarin solution). To improve lifetime fits, a binning factor of one (i.e., 3×3 neighboring pixels) was applied to the decays. The instrument response was deconvolved from the raw decay measurements through an iterative reconvolution fitting algorithm that performs a least-squares minimization of the residuals. To determine NAD(P)H fluorescence lifetime parameters, the pixel decays were fit to a biexponential model: $I(t)=\alpha_{1}e^{-t/\tau_{1}}+\alpha_{2}e^{-t/\tau_{2}}$. Mean fluorescence lifetime was estimated as $\tau_{m}=\alpha_{1}\tau_{1}+\alpha_{2}\tau_{2}$.

For visualization purposes, the NAD(P)H lifetime images from the SPAD camera were upscaled using the intensity image from the sCMOS camera acquired through the 530/55  nm bandpass filter with a 1 s integration time. For upscaling, the SPAD lifetime images were interpolated to the resolution of the CMOS image (i.e., 4 times higher pixel count) and weighted by the CMOS pixel intensities. For live *in vivo* imaging of mCherry-labeled neutrophils, masks were generated from the mCherry image using a binary threshold in ImageJ,^55^ and this mask was applied to the NAD(P)H FLIM image.

### 2.5 Sample Preparation: In Vitro Pancreatic Cancer Cells

PANC-1 human pancreatic cancer cells (ATCC) were maintained in high-glucose DMEM supplemented with 10% fetal bovine serum and 1% penicillin/streptomycin. Cells were pelleted, re-suspended in culture medium supplemented with 2% agarose and loaded into FEP tubing suspended in a water bath (Fig. 1). Known metabolic perturbations were induced by exposing cells to sodium cyanide (1 mM), which binds to electron transport chain complex IV and inhibits aerobic ATP production; this in turn increases the amount of free NAD(P)H in the cell, detectable as a decrease in mean fluorescence lifetime and an increase in fluorescence intensity.^56^^–^^58^ NAD(P)H FLIM images of live PANC-1 cells were acquired every 3 min using the SPAD camera and a 440/80  nm filter with an integration time of 10 s to ensure sufficient photon counts (>500  photons per pixel before binning). An NAD(P)H intensity image was also acquired using the sCMOS camera and a 530/55  nm filter with a 1 s integration time to upscale the SPAD FLIM images for better visualization.

### 2.6 Sample Preparation: In Vivo Zebrafish Tail Wound Model

Animal care and use was approved by the Institutional Animal Care and Use Committee of University of Wisconsin and strictly followed guidelines set by the federal Health Research Extension Act and the Public Health Service Policy on the Humane Care and Use of Laboratory Animal, administered by the National Institute of Health Office of Laboratory Animal Welfare.

All protocols using zebrafish in this study have been approved by the University of Wisconsin-Madison Research Animals Resource Center (protocols M005405-A02). Adult zebrafishes were maintained on a 14/10 h light/dark schedule. Upon fertilization, embryos were transferred into E3 medium (5 mM NaCl, 0.17 mM KCl, 0.44 mM ${\mathrm{CaCl}}_{2}$, 0.33 mM ${\mathrm{MgSO}}_{4}$, 0.025 mM NaOH, and 0.0003% methylene blue) and maintained at 28.5°C. Adult “casper” fish^59^ and wild-type transgenic Tg(mpx:mCherry) in AB background^60^ were used in this study. Tg(mpx:mCherry) fishes were outcrossed to casper fish to generate transgenic fish without pigmentation. Larvae at 3 days post-fertilization (dpf) were screened for mCherry expression using a ZEISS Axio Zoom.V16 fluorescence stereo zoom microscope (EMS3/SyCoP3, Zeiss: Plan-Neofluar Z 1X:0.25 FWD 56-mm lens) and raised to breeding age. These adult fishes were used for incross breeding. Larvae at 3 dpf were screened for casper larvae with mCherry expression as a cytoplasmic marker of neutrophils, which are dynamic innate immune cells that rapidly migrate to wounds during early healing stages.^60^^,^^61^

3 dpf larvae were anesthetized in E3 medium containing 0.16  mg/mL Tricaine (ethyl 3-aminobenzoate; Sigma-Aldrich) and caudal fin transection was performed^11^ 30 min prior to imaging. Larvae were mounted in a 0.8-mm inner diameter FEP tube in 0.4% low-melting-point agarose with 0.16  mg/ml Tricaine and an agarose plug. We collected brightfield (25 ms exposure sCMOS), mCherry intensity (1 s exposure sCMOS), and NAD(P)H lifetime (10 s integration SPAD) from neutrophils in quick succession (every 2 min). NAD(P)H FLIM was acquired in a z-stack with four slices through the thickness of the tail fin. The sCMOS mCherry image was superimposed on the NAD(P)H fluorescence lifetime image to generate neutrophil masks, and lifetime information was extracted from single cells.

## 3 Results

### 3.1 System Characterization and Calibration

**Fig. 2:**
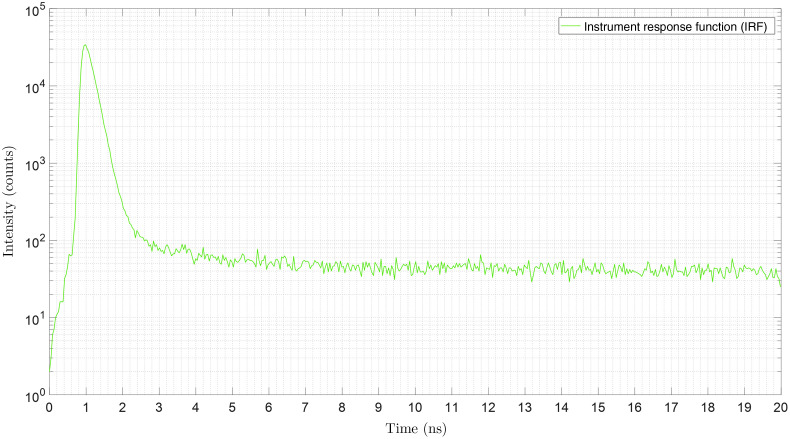
IRF. To measure the combined IRF of the excitation laser (Omicron QuixX 375-70PS) and the SPAD camera (Horiba FLIMera), a fraction of the excitation laser pulses (50 MHz pulse repetition rate) was directly imaged onto the FLIMera camera by imaging a retroreflector target in an epi-illuminated widefield microscope setup. The 20-ns time axis consists of 486 time bins of 41.1 ps width. The IRF has an FWHM of 380 ps. The DCR of individual pixels can be measured from the non-zero tail of the pixels’ IRF.

The combined IRF of the 50 MHz pulsed laser source and the SPAD array camera is shown in Fig. 2. The system IRF has an FWHM of 380 ps. Due to non-zero DCRs of the SPAD pixels, the measured IRF drops to a non-zero constant in later time bins. This tail of the measured IRF was used to estimate per-pixel DCRs. A histogram of the estimated DCRs for this SPAD camera is shown in Fig. S1 in the Supplementary Material. The median DCR of the SPAD pixels was 34 cps.

**Fig. 3:**
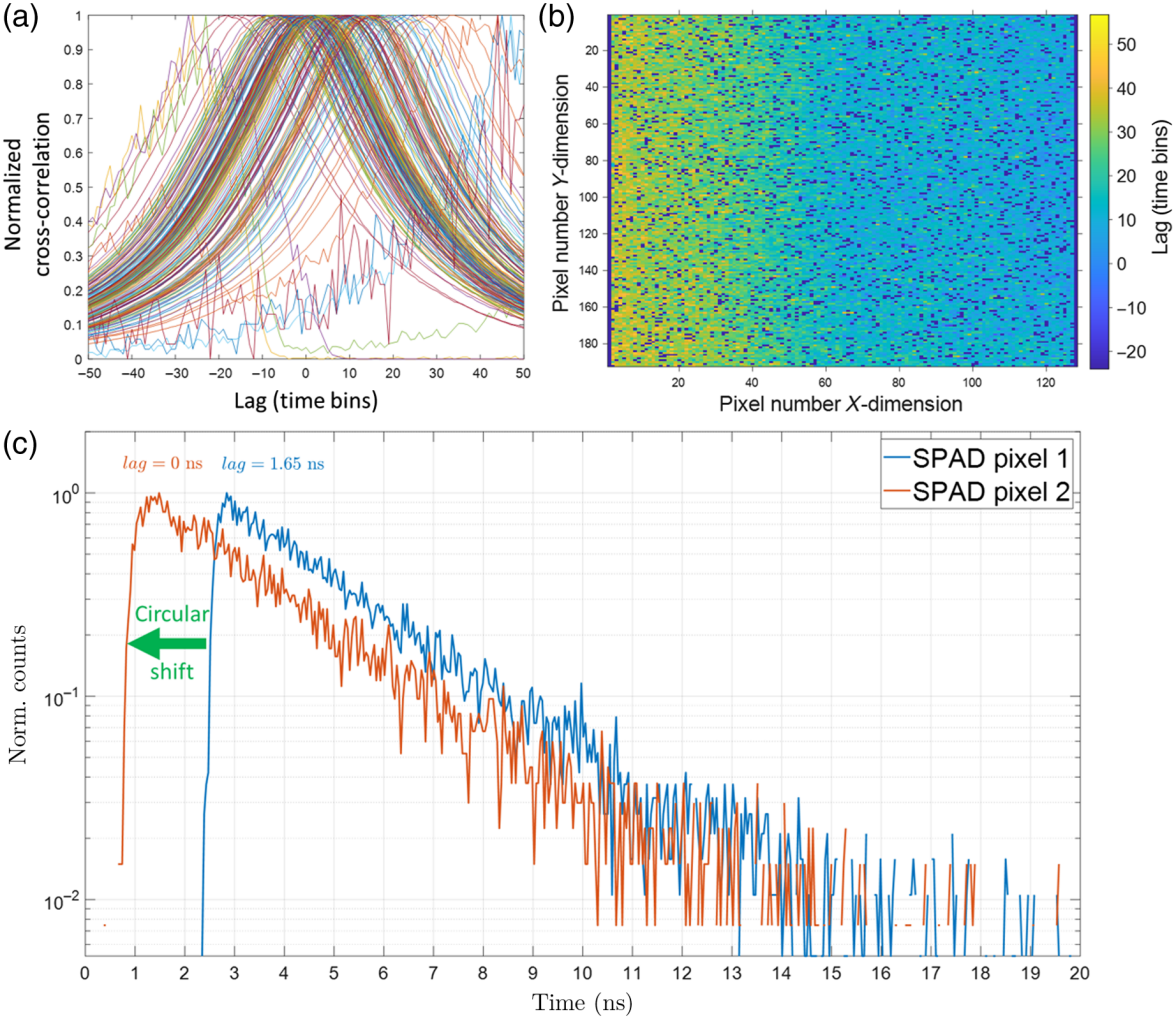
FLIMera SPAD array camera inter-pixel delay map. To compensate for the inter-pixel timing skew and synchronize the rise time of all 192×128  pixels, the normalized cross-correlation of the measured decay for each pixel with a reference decay was calculated. (a) These cross-correlation curves for a representative group of SPAD pixels (each curve is one pixel). The temporal lag value where the normalized cross-correlation is maximized was recorded and mapped as seen in (b) for every pixel of the SPAD array. The “screamer” dead pixels are dark blue and comprise 15% of all pixels. The inter-pixel lag between pixels on the left and right side of the sensor can be as high as 60 time bins (with 41.1 ps time bin width) or ∼2.5  ns, which is substantial. To synchronize the rise time of all pixels, it suffices to apply an opposite circular shift to the decay histogram of each SPAD pixel by the amount given by the delay map above. (c) The decays from two representative SPAD pixels that have different timing lags. SPAD pixel 1 has a lag of 40 time bins or 1.65 ns with respect to the reference decay, whereas SPAD pixel 2 has a lag of zero. To correct the timing skew, the blue curve should be shifted to the left by 40 time bins.

**Fig. 4:**
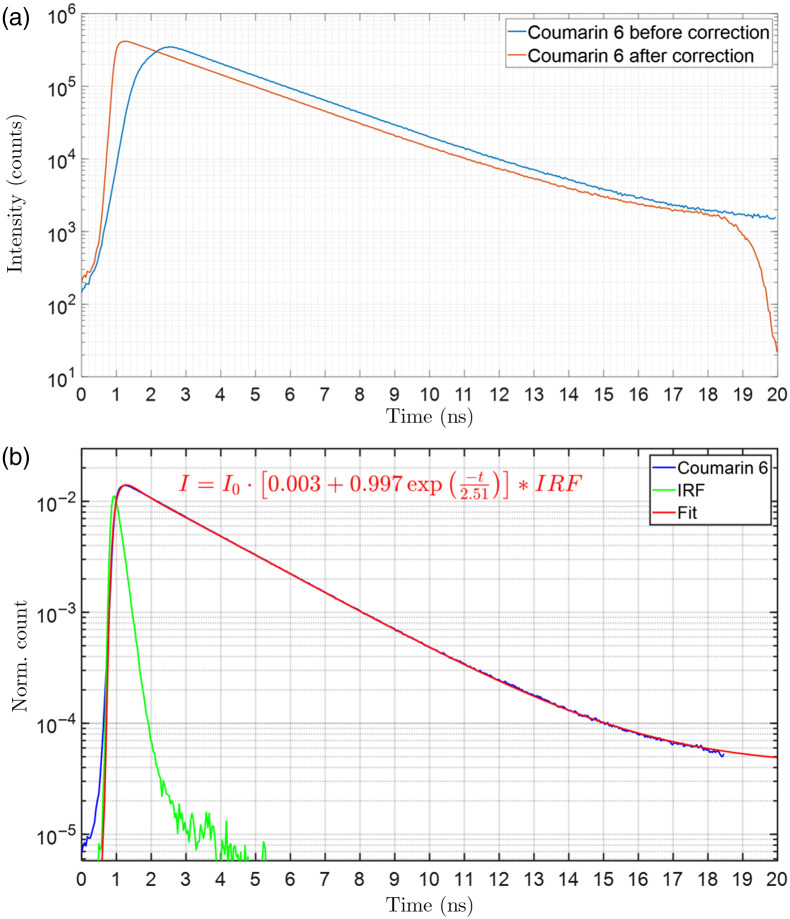
Fluorescence lifetime fitting of coumarin 6 in ethanol. A saturated solution of coumarin 6 in ethanol was loaded into an FEP sample tube and imaged on the light-sheet SPAD system with 0.4 mW excitation laser power and 3 s integration time using a green emission filter (550/100  nm). (a) The blue curve shows the aggregated decay histogram from all array pixels before correcting for the inter-pixel delays. The red curve shows the aggregated decay histogram after such correction. The corrected decay histogram has a faster rise and higher peak, whereas the uncorrected decay histogram has a slower rise and is temporally broadened. (b) The aggregated decay histogram of all pixels after timing skew correction was fit to a single-exponential decay model using an iterative reconvolution algorithm that performs a least-squares minimization of the residuals. A fluorescence lifetime of 2.51 ns is estimated which agrees with reported published values.^62^^–^^64^

A map of the inter-pixel delays across the SPAD array sensor was estimated from the coumarin 6 fluorescence lifetime image as shown in Fig. 3 using a pixel-wise cross-correlation maximization scheme. If left uncorrected, this non-uniform delay results in distortion artifacts when aggregating decays from different pixels on the sensor (e.g., for pixel binning during lifetime fit analysis). Figure 4 shows the effect of correcting for these inter-pixel delays on the aggregate decay histogram of coumarin 6. The estimated fluorescence lifetime after such correction (2.51 ns) agrees with reported values in the literature.^62^^–^^64^ Figures S2 and S3 in the Supplementary Material show lifetime images and aggregate decays for YG and BB fluorescent microspheres. The estimated lifetime of 2.15 ns for YG beads and 2.6 ns for BB beads also agree with reported values in the literature.^65^^,^^66^

### 3.2 Effects of Cyanide on NAD(P)H Metabolism In Vitro

**Fig. 5:**
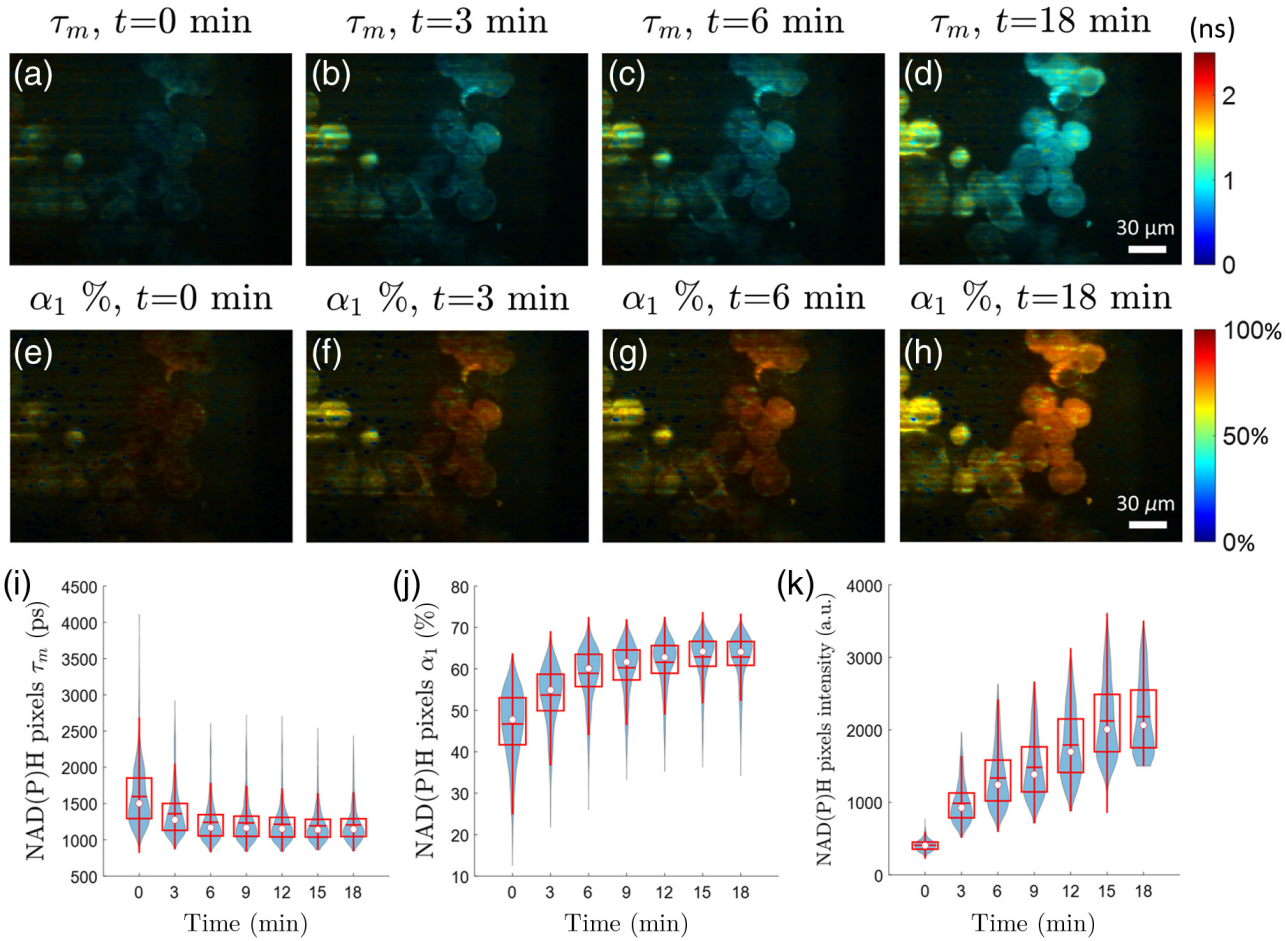
Cyanide treatment of PANC-1 cells confirms the sensitivity of the light-sheet FLIM system to NAD(P)H fluorescence lifetime changes (Video 1, MPEG, 0.5 MB [URL: https://doi.org/10.1117/1.JBO.28.6.066502.s1]). (a)–(d) NAD(P)H mean fluorescence lifetime ($\tau_{m}$) images of PANC-1 cells at different time points after treatment with 1 mM sodium cyanide. The sCMOS NAD(P)H intensity image is used to upscale the SPAD array NAD(P)H lifetime image (e)–(h). Corresponding free fraction of NAD(P)H ($\alpha_{1}$) at the same time points. (i) Boxplot of NAD(P)H $\tau_{m}$ of image pixels over time shows a rapid drop in mean lifetime within a few minutes of cyanide treatment. (j) NAD(P)H $\alpha_{1}$ of image pixels increases over time. (k) The pixel intensity of NAD(P)H fluorescence, measured by integrating the decay curve at each pixel in the SPAD camera, increases over time with cyanide exposure. White dot shows the median; red horizontal line shows the mean; box encompasses 25th to 75th percentile range; whiskers extend from the box to 1.5 times the interquartile range. Changes in NAD(P)H mean lifetime (F-statistic = 9.78, p=0.026), free fraction (F-statistic = 21.8, p=0.006), and mean intensity (F-statistic = 113, p=0.0001) with time are significant according to the linear trend test.

After the addition of 1 mM cyanide, NAD(P)H FLIM images of PANC-1 cells were taken every 3 min (Fig. 5). We observed the expected decrease in NAD(P)H mean fluorescence lifetime [$\tau_{m}$, Figs. 5(a)–5(d) and 5(i)], an increase in the free fraction of NAD(P)H [$\alpha_{1}$, Figs. 5(e)–5(h) and 5(j)], and an increase in the NAD(P)H fluorescence intensity [Fig. 5(k)], starting at 3 min post-exposure and continuing over time. These changes in NAD(P)H intensity and lifetime parameters were expected,^4^^,^^56^^,^^58^^,^^67^ and the range of NAD(P)H $\tau_{m}$ and $\alpha_{1}$ also fall within published values for cells in culture.^57^^,^^68^ These experiments confirm the sensitivity of this system to NAD(P)H lifetimes and to physiologically relevant changes in NAD(P)H lifetimes. These studies also demonstrate that this NAD(P)H light-sheet FLIM system can perform time-course imaging of single-cell autofluorescence in a fluorescent background of culture medium. Further, we found that 10 s integration time was sufficient to capture enough NAD(P)H photons for accurate lifetime fitting analysis.

### 3.3 Light-Sheet FLIM of NAD(P)H In Vivo in Larval Zebrafish

**Fig. 6:**
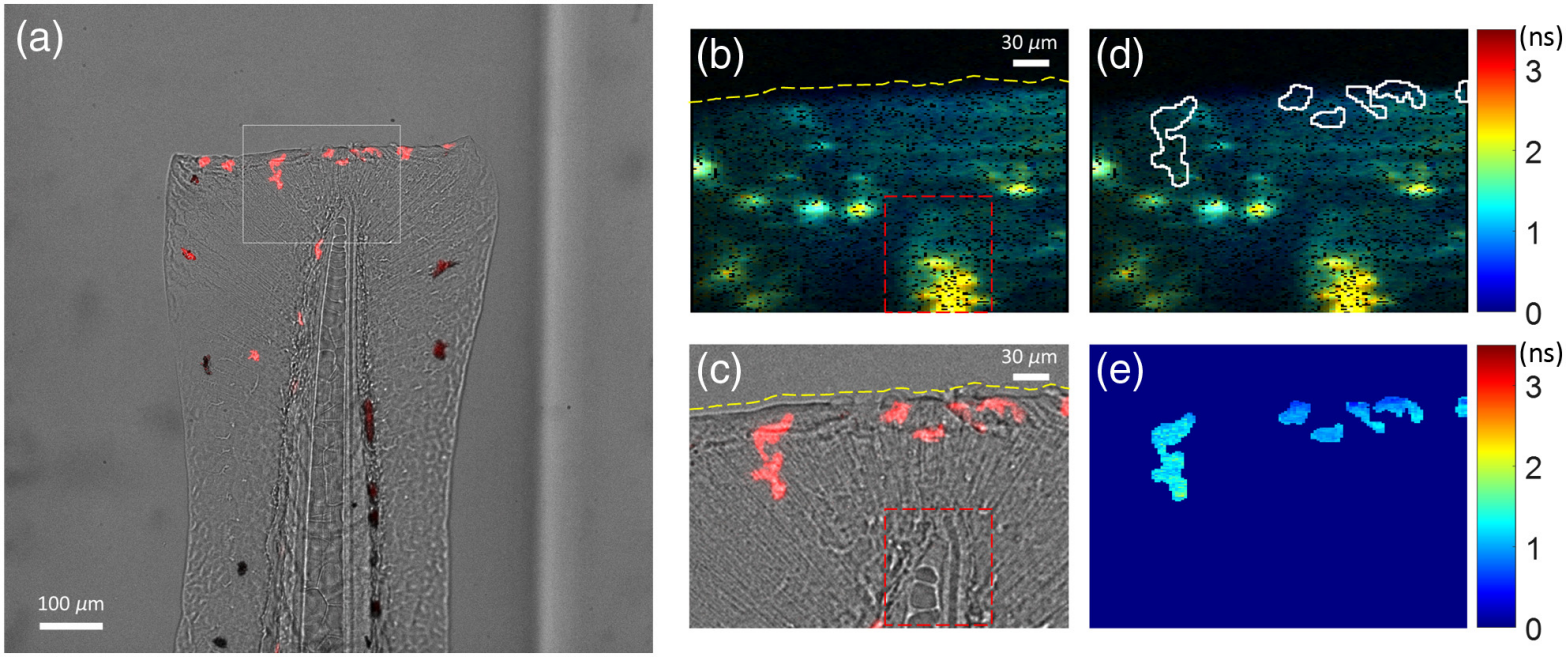
Light-sheet imaging of mCherry intensity and NAD(P)H lifetimes in live neutrophils *in vivo* in a zebrafish caudal fin wound model. (a) The intensity channel of the light-sheet FLIM system shows the superimposed brightfield image of 3 dpf wounded zebrafish tail and light-sheet-excited fluorescence intensity of mCherry+ neutrophils at the wound site (Video 2, MPEG, 2.1 MB [URL: https://doi.org/10.1117/1.JBO.28.6.066502.s2]). The white rectangle shows the corresponding field of view of the smaller SPAD array sensor. (b) NAD(P)H mean fluorescence lifetime ($\tau_{m}$) image of the tail (from the SPAD array with 10 s integration). The transection wound (yellow dashed line) and the tip of the notochord (red dashed box) are visible in the lifetime image. (c) Corresponding field of view from the cropped sCMOS image shows mCherry+ neutrophils recruited to the wound site. (d) Pixel mask of neutrophil cells from the mCherry channel applied to the NAD(P)H lifetime image. (e) Masked NAD(P)H lifetime map of the neutrophils.

We next sought to measure NAD(P)H lifetime in a dynamic, *in vivo* environment (Fig. 6). NAD(P)H is present in all cell types of a living organism, so we used a zebrafish caudal fin wound model to track mCherry-labeled neutrophils,^69^ innate immune cells recruited to the injury site. To demonstrate light-sheet NAD(P)H FLIM in these dynamic cells, an mCherry intensity image was acquired using light-sheet excitation from the left (CW) illumination arm whose sectioning plane was aligned to perfectly match the right (pulsed) illumination arm that excites NAD(P)H for the FLIM image. NAD(P)H FLIM was simultaneously acquired in a z-stack to track the same neutrophils over time. The mCherry fluorescence was used to identify the cells of interest and create masks to isolate NAD(P)H specifically from the neutrophils, as autofluorescence signal is generated from the whole tail fin tissue in the FOV. These studies demonstrate that the NAD(P)H light-sheet FLIM system is sensitive to single cells *in vivo*, and that combined intensity and FLIM images can be used to isolate quickly moving cells *in vivo*.

## 4 Discussion and Conclusion

Detector arrays in a widefield geometry provide faster FLIM acquisition speed (i.e., shorter integration time per frame) compared to a single-detector multiphoton or confocal laser scanning geometry. Recent developments in SPAD array sensor technology enable widefield FLIM with high TCSPC temporal resolution. These new SPAD cameras can be combined with low-cost pulsed diode lasers for rapid FLIM. Table 1 shows the theoretical advantage in integration time for the SPAD array used in this work over a standard laser scanning FLIM microscope using a single TCSPC module for acquiring an image of the same size. However, the simplest implementation of widefield FLIM, i.e., an epi-fluorescence geometry, can only be applied to bright fluorescently labeled samples due to lack of optical sectioning. In label-free applications, out-of-focus background fluorescence can overwhelm the weak autofluorescence signals from single cells. Thus widefield autofluorescence FLIM has remained impractical.

**Table 1:** Comparison of theoretical FLIM imaging speed limit between laser scanning and widefield geometries. The saturation count rate for the SPAD camera with per-pixel TDC modules working in parallel is 30× higher than the saturation count rate of a single TCSPC module in laser scanning microscopes. The speed advantage scales with the number of array pixels.

	Laser scanning (PMT + TAC)	Widefield (SPAD array + TDC)
Image pixel count	192×128=∼25,000	192×128=∼25,000
	Sequential	Simultaneous
Detector dead time (ns)	∼100 ns	∼80,000 ns
Max pixel count rate (cps)	$∼10\times 10^{6}$	∼12,000
Max system count rate (cps)	$∼10\times 10^{6}$	$∼300\times 10^{6}$
Effective pixel count rate (cps)	∼400	∼12,000

In this work, we combined multichannel detection using a SPAD array camera with light-sheet excitation to minimize the effects of out-of-focus background fluorescence. In addition to providing optical sectioning, light-sheet illumination is more efficient at exciting fluorophores than epi-illumination because photons traveling in the focus plane have a higher probability of encountering a fluorophore. The peak irradiance of a light sheet is also many orders of magnitude lower than a diffraction-limited spot in laser scanning (e.g., confocal) microscopy, which decreases phototoxicity. Artifacts are noticeable in areas of the presented lifetime images where dead “screamer” SPAD pixels (that comprise 15% of all pixels) cluster together. We also acknowledge that a secondary streaking artifact is visible in Figs. 5 and 6 due to shadowing in the light-sheet excitation path. This is caused by absorption in the sample and can be mitigated by pivoting the light sheet (also known as dithering) such that these shadows are averaged out during the integration period.^52^ Such artifacts could be eliminated in future designs using UV-optimized multidirectional/pivoting optics.

The low quantum yield of endogenous fluorophores [e.g., NAD(P)H, FAD]^18^ limits the acquisition speed of autofluorescence FLIM because photodamage to cells occurs before the detector count rate saturates. As such, it is helpful to compare the light-sheet SPAD system to a laser scanning system for the imaging settings used and count rates observed for NAD(P)H FLIM in practice. This comparison is made in Table 2 for the specific 2P laser scanning microscope,^67^ the light-sheet SPAD system, and the PANC-1 cells used in this study. The pixel count rate and photons per pixel are reported before any pixel binning. Figure S4 in the Supplementary Material shows a representative two-photon FLIM image of PANC-1 cells. For the same field of view, number of image pixels, and targeted photon count per pixel, the light-sheet SPAD system in Fig. 1 requires 6× less integration time to acquire the NAD(P)H image compared to a 2P laser scanning microscope. Frame rates of SPAD arrays relative to single-pixel laser scanning systems scale with the number of array pixels, when acquiring the same number of image photons. SPAD arrays are based on the same CMOS fabrication process as existing cameras, so the commercial infrastructure for inexpensive and large-scale manufacturing of high-resolution arrays exists.^70^ This has resulted in a rapid improvement in the capabilities of SPAD systems with improved photon detection probabilities and fill factors (e.g., using microlens arrays over SPAD pixels^71^^,^^72^), which can further reduce integration time per frame in FLIM or reduce excitation light dose for reduced phototoxicity over long imaging sessions, which is an important consideration given the higher phototoxicity of shorter wavelengths.^45^

**Table 2:** Comparison of practical FLIM imaging speed limit between a standard 2P laser scanning system and the light-sheet system in Fig. 1. For the same field of view size, number of image pixels, and targeted number of emission photons per image pixel, the light-sheet system required 6× less integration time per image.

	2P laser scanning (PMT + TAC)	1P light sheet (SPAD array + TDC)
Laser power	5 mW at 750 nm	0.4 mW at 375 nm
	Ø1 μm diffraction-limited	200 μm×9 μm sheet
	Obj. 40×/1.15 NA	Obj. 10×/0.3 NA
Peak irradiance ($W/{\mathrm{cm}}^{2}$)	∼640,000	∼28
FOV	175 μm×235 μm	175 μm×235 μm
Image pixel count	192×128=∼25,000 sequential	192×128=∼25,000 simultaneous
Time resolution (ps)	39	41
Total integration time (s)	60	10
Pixel dwell time	2.4 ms	10 s
Pixel photon count rate (cps)	$∼1.3\times 10^{6}$	∼300
Photons per pixel	∼3000	∼3000
Effective pixel count rate (cps)	∼50	∼300
Light dose	$∼940\;J/{\mathrm{cm}}^{2}$ at 750 nm	$∼280\;J/{\mathrm{cm}}^{2}$ at 375 nm

Existing light-sheet FLIM microscopes perform well for samples with bright fluorophores, but access to weaker autofluorescence lifetimes is challenging due to limitations in sensitivity and temporal resolution of current detector arrays. Here, we show that it is feasible to use a commercial SPAD array in a light-sheet microscope for NAD(P)H FLIM of single cells *in vitro* and *in vivo*. The integration time per image for this configuration is 6× faster than that of traditional laser scanning NAD(P)H FLIM microscopes. Faster acquisition speeds will be achieved as SPAD arrays continue to improve in detection efficiency and array size.

The FLIM frame interval in these studies was limited by write-to-disk speed when saving data in raw photon stream mode, which imposed delays between successive frames. Specifically, the FLIMera camera can only transfer data in photon streaming mode over USB 3.0, with histogram compilation and lifetime analysis on the computer CPU. The process of transferring individual photon time tags costs both USB data transfer bandwidth and analysis time, which prohibits the acquisition of successive frames at the maximum speed of the hardware. Some SPAD array developers use on-chip histogramming with an FPGA^73^ to substantially reduce this data transfer and analysis bottleneck, enabling larger SPAD arrays with per-pixel TDC. Alternatively, a time-gating scheme with a user-selectable number and width of time gates can be used instead of TCSPC. This approach removes per-pixel TDC electronics and simplifies the design to provide larger SPAD arrays with better fill factors. This approach also allows the user to trade temporal resolution for imaging speed by reducing the number of time gates. For example, time gating was employed in SwissSPAD detectors with 512×512  pixels.^38^ Therefore, as sensor sensitivity improves, parallel improvements in data streaming and analysis will be necessary to reach the full frame-rate potential of SPAD arrays for NAD(P)H light-sheet FLIM.

Overall, these NAD(P)H light-sheet FLIM systems will be an important new tool to study single cell metabolism and migration in 3D, including *in vivo* studies of whole model organisms.

## Data Availability

All data and code used in the analyses are available for purposes of reproducing or extending the analyses through a GitHub repository (https://github.com/skalalab/Light-sheet-SPAD-array).

## References

[1] Cuny A. P. et al., “Live cell microscopy: from image to insight,” Biophys. Rev. 3, 021302 (2022).1793-0480 10.1063/5.0082799 PMC10903399 38505412

[2] Datta R. et al., “Fluorescence lifetime imaging microscopy: fundamentals and advances in instrumentation, analysis, and applications,” J. Biomed. Opt. 25, 071203 (2020). JBOPFO 1083-3668 10.1117/1.JBO.25.7.071203 32406215 PMC7219965

[r3] Heaster T. M. et al., “Autofluorescence imaging of 3D tumor-macrophage microscale cultures resolves spatial and temporal dynamics of macrophage metabolism,” Cancer Res. 80, 5408–5423 (2020). CNREA8 0008-5472 10.1158/0008-5472.CAN-20-0831 33093167 PMC7718391

[r4] Shah A. T. et al., “In vivo autofluorescence imaging of tumor heterogeneity in response to treatment,” Neoplasia-U. S. 17, 862–870 (2015). 10.1016/j.neo.2015.11.006 PMC4688562 26696368

[r5] Walsh A. J. et al., “Quantitative optical imaging of primary tumor organoid metabolism predicts drug response in breast cancer,” Cancer Res. 74, 5184–5194 (2014). CNREA8 0008-5472 10.1158/0008-5472.CAN-14-0663 25100563 PMC4167558

[r6] Walsh A. J. et al., “Optical imaging of drug-induced metabolism changes in murine and human pancreatic cancer organoids reveals heterogeneous drug response,” Pancreas 45, 863–869 (2016). PANCE4 0885-3177 10.1097/MPA.0000000000000543 26495796 PMC4874911

[r7] Sharick J. T. et al., “Metabolic heterogeneity in patient tumor-derived organoids by primary site and drug treatment,” Front. Oncol. 10, 553 (2020). FRTOA7 0071-9676 10.3389/fonc.2020.00553 32500020 PMC7242740

[r8] Walsh A. J. et al., “Classification of T-cell activation via autofluorescence lifetime imaging,” Nat. Biomed. Eng. 5, 77–88 (2021). 10.1038/s41551-020-0592-z 32719514 PMC7854821

[r9] Chance B. et al., “Oxidation-reduction ratio studies of mitochondria in freeze-trapped samples. NADH and flavoprotein fluorescence signals,” J. Biol. Chem. 254, 4764–4771 (1979). JBCHA3 0021-9258 10.1016/S0021-9258(17)30079-0 220260

[r10] Sharick J. T. et al., “Protein-bound NAD(P)H lifetime is sensitive to multiple fates of glucose carbon,” Sci. Rep. 8, 5456 (2018). SRCEC3 2045-2322 10.1038/s41598-018-23691-x 29615678 PMC5883019

[r11] Miskolci V. et al., “In vivo fluorescence lifetime imaging of macrophage intracellular metabolism during wound responses in zebrafish,” eLife 11, e66080 (2022). 10.7554/eLife.66080 35200139 PMC8871371

[r12] Schmitz R. et al., “Extracellular pH affects the fluorescence lifetimes of metabolic co-factors,” J. Biomed. Opt. 26, 056502 (2021). JBOPFO 1083-3668 10.1117/1.JBO.26.5.056502 34032035 PMC8144436

[13] Qian T. et al., “Label-free imaging for quality control of cardiomyocyte differentiation,” Nat. Commun. 12, 4580 (2021). NCAOBW 2041-1723 10.1038/s41467-021-24868-1 34321477 PMC8319125

[14] Lakowicz J. R. et al., “Fluorescence lifetime imaging of free and protein-bound NADH,” Proc. Natl. Acad. Sci. U. S. A. 89, 1271–1275 (1992). 10.1073/pnas.89.4.1271 1741380 PMC48431

[15] Walsh A. J. Skala M. C., “An automated image processing routine for segmentation of cell cytoplasms in high-resolution autofluorescence images,” Proc. SPIE 8948, 89481M (2014). PSISDG 0277-786X 10.1117/12.2040644

[16] Qin Y. Xia Y., “Simultaneous two-photon fluorescence microscopy of NADH and FAD using pixel-to-pixel wavelength-switching,” Front. Phys. 9, 642302 (2021). 10.3389/fphy.2021.642302

[17] Zipfel W. R. Williams R. M. Webb W. W., “Nonlinear magic: multiphoton microscopy in the biosciences,” Nat. Biotechnol. 21, 1369–1377 (2003). NABIF9 1087-0156 10.1038/nbt899 14595365

[18] Kasischke K. A. et al., “Neural activity triggers neuronal oxidative metabolism followed by astrocytic glycolysis,” Science 305, 99–103 (2004). SCIEAS 0036-8075 10.1126/science.1096485 15232110

[r19] Scott T. G. et al., “Synthetic spectroscopic models related to coenzymes and base pairs. V. Emission properties of NADH. Studies of fluorescence lifetimes and quantum efficiencies of NADH, AcPyADH, [reduced acetylpyridineadenine dinucleotide] and simplified synthetic models,” J. Am. Chem. Soc. 92, 687–695 (1970). JACSAT 0002-7863 10.1021/ja00706a043

[20] Follenius-Wund A. et al., “Fluorescent derivatives of the GFP chromophore give a new insight into the GFP fluorescence process,” Biophys. J. 85, 1839–1850 (2003). BIOJAU 0006-3495 10.1016/S0006-3495(03)74612-8 12944297 PMC1303356

[21] Bruschini C. et al., “Single-photon avalanche diode imagers in biophotonics: review and outlook,” Light Sci. Appl. 8, 87–87 (2019). 10.1038/s41377-019-0191-5 31645931 PMC6804596

[22] Singh A. P. et al., “The performance of 2D array detectors for light sheet based fluorescence correlation spectroscopy,” Opt. Express 21, 8652–8652 (2013). OPEXFF 1094-4087 10.1364/OE.21.008652 23571955

[r23] Michalet X. et al., “Detectors for single-molecule fluorescence imaging and spectroscopy,” J. Mod. Opt. 54, 239–281 (2007). JMOPEW 0950-0340 10.1080/09500340600769067 20157633 PMC2821066

[24] Vitali M. et al., “A single-photon avalanche camera for fluorescence lifetime imaging microscopy and correlation spectroscopy,” IEEE J. Sel. Top. Quantum Electron. 20, 344–353 (2014). IJSQEN 1077-260X 10.1109/JSTQE.2014.2333238

[25] Madonini F. Villa F., “Single photon avalanche diode arrays for time-resolved Raman spectroscopy,” Sensors 21, 4287–4287 (2021). SNSRES 0746-9462 10.3390/s21134287 34201576 PMC8272195

[r26] Kostamovaara J. et al., “Fluorescence suppression in Raman spectroscopy using a time-gated CMOS SPAD,” Opt. Express 21, 31632–31632 (2013). OPEXFF 1094-4087 10.1364/OE.21.031632 24514736

[27] Nissinen I. et al., “A 16 × 256 SPAD line detector with a 50-ps, 3-bit, 256-channel time-to-digital converter for Raman spectroscopy,” IEEE Sens. J. 18, 3789–3798 (2018). ISJEAZ 1530-437X 10.1109/JSEN.2018.2813531

[28] Williams G. O. S. et al., “Full spectrum fluorescence lifetime imaging with 0.5 nm spectral and 50 ps temporal resolution,” Nat. Commun. 12, 6616 (2021). NCAOBW 2041-1723 10.1038/s41467-021-26837-0 34785666 PMC8595732

[29] Tanner M. G. et al., “Ballistic and snake photon imaging for locating optical endomicroscopy fibres,” Biomed. Opt. Express 8, 4077–4077 (2017). BOEICL 2156-7085 10.1364/BOE.8.004077 28966848 PMC5611924

[r30] Stuker F. et al., “Hybrid small animal imaging system combining magnetic resonance imaging with fluorescence tomography using single photon avalanche diode detectors,” IEEE Trans. Med. Imaging 30, 1265–1273 (2011). ITMID4 0278-0062 10.1109/TMI.2011.2112669 21317083

[31] Mora A. D. et al., “Towards next-generation time-domain diffuse optics for extreme depth penetration and sensitivity,” Biomed. Opt. Express 6, 1749–1749 (2015). BOEICL 2156-7085 10.1364/BOE.6.001749 26137377 PMC4467698

[32] Poland S. P. et al., “Time-resolved multifocal multiphoton microscope for high speed FRET imaging in vivo,” Opt. Lett. 39, 6013 (2014). OPLEDP 0146-9592 10.1364/OL.39.006013 25361143

[r33] Poland S. P. et al., “A high speed multifocal multiphoton fluorescence lifetime imaging microscope for live-cell FRET imaging,” Biomed. Opt. Express 6, 277 (2015). BOEICL 2156-7085 10.1364/BOE.6.000277 25780724 PMC4354599

[r34] Poland S. P. et al., “Multifocal multiphoton volumetric imaging approach for high-speed time-resolved Förster resonance energy transfer imaging in vivo,” Opt. Lett. 43, 6057 (2018). OPLEDP 0146-9592 10.1364/OL.43.006057 30548010 PMC6410918

[35] Levitt J. A. et al., “Quantitative real-time imaging of intracellular FRET biosensor dynamics using rapid multi-beam confocal FLIM,” Sci. Rep. 10, 5146 (2020). SRCEC3 2045-2322 10.1038/s41598-020-61478-1 32198437 PMC7083966

[36] Burri S. et al., “Architecture and applications of a high resolution gated SPAD image sensor,” Opt. Express 22, 17573–17573 (2014). OPEXFF 1094-4087 10.1364/OE.22.017573 25090572 PMC4162351

[37] Schwartz D. E. Charbon E. Shepard K. L., “A single-photon avalanche diode array for fluorescence lifetime imaging microscopy,” IEEE J. Solid-State Circuits 43, 2546–2557 (2008). IJSCBC 0018-9200 10.1109/JSSC.2008.2005818 23976789 PMC3748627

[38] Ulku A. C. et al., “A 512 × 512 SPAD image sensor with integrated gating for widefield FLIM,” IEEE J. Sel. Top. Quantum Electron. 25, 1–12 (2019). IJSQEN 1077-260X 10.1109/JSTQE.2018.2867439 PMC6541425 31156324

[39] Zickus V. et al., “Fluorescence lifetime imaging with a megapixel SPAD camera and neural network lifetime estimation,” Sci. Rep. 10, 20986 (2020). SRCEC3 2045-2322 10.1038/s41598-020-77737-0 33268900 PMC7710711

[40] Li D.-U. et al., “Real-time fluorescence lifetime imaging system with a 32×32 013 μm CMOS low dark-count single-photon avalanche diode array,” Opt. Express 18, 10257–10257 (2010). OPEXFF 1094-4087 10.1364/OE.18.010257 20588879

[41] Stewart H. L. Hungerford G. Birch D. J. S., “Characterization of single channel liquid light guide coupling and SPAD array imaging for tumour margin estimation using fluorescence lifetime,” Meas. Sci. Technol. 31, 125701 (2020). MSTCEP 0957-0233 10.1088/1361-6501/aba5c6

[42] Li D. D.-U. et al., “Time-domain fluorescence lifetime imaging techniques suitable for solid-state imaging sensor arrays,” Sensors 12, 5650–5669 (2012). SNSRES 0746-9462 10.3390/s120505650 22778606 PMC3386705

[43] Homulle H. A. R. et al., “Compact solid-state CMOS single-photon detector array for in vivo NIR fluorescence lifetime oncology measurements,” Biomed. Opt. Express 7, 1797 (2016). BOEICL 2156-7085 10.1364/BOE.7.001797 27231622 PMC4871082

[44] Huisken J. Stainier D. Y. R., “Selective plane illumination microscopy techniques in developmental biology,” Development 136, 1963–1975 (2009). 10.1242/dev.022426 19465594 PMC2685720

[r45] Wagner M. et al., “Light dose is a limiting factor to maintain cell viability in fluorescence microscopy and single molecule detection,” Int. J. Mol. Sci. 11, 956–966 (2010).1422-0067 10.3390/ijms11030956 20479994 PMC2869222

[46] Hillman E. M. C. et al., “Light-sheet microscopy in neuroscience,” Annu. Rev. Neurosci. 42, 295–313 (2019). ARNSD5 0147-006X 10.1146/annurev-neuro-070918-050357 31283896 PMC6800245

[47] Hirvonen L. M. et al., “Lightsheet fluorescence lifetime imaging microscopy with wide‐field time‐correlated single photon counting,” J. Biophotonics 13, e201960099 (2020). 10.1002/jbio.201960099 31661595 PMC7065631

[48] Mitchell C. A. et al., “Functional in vivo imaging using fluorescence lifetime light-sheet microscopy,” Opt. Lett. 42, 1269–1269 (2017). OPLEDP 0146-9592 10.1364/OL.42.001269 28362747

[49] Li R. et al., “Digital scanned laser light‐sheet fluorescence lifetime microscopy with wide‐field time‐gated imaging,” J. Microsc. 279, 69–76 (2020). JMICAR 0022-2720 10.1111/jmi.12898 32307699

[50] Greger K. et al., “Three-dimensional fluorescence lifetime imaging with a single plane illumination microscope provides an improved signal to noise ratio,” Opt. Express 19, 20743 (2011). OPEXFF 1094-4087 10.1364/OE.19.020743 21997084

[51] Henderson R. K. et al., “A 192 × 128 time correlated SPAD image sensor in 40-nm CMOS technology,” IEEE J. Solid-State Circuits 54, 1907–1916 (2019). 10.1109/JSSC.2019.2905163

[52] Huisken J. Stainier D. Y. R., “Even fluorescence excitation by multidirectional selective plane illumination microscopy (mSPIM),” Opt. Lett. 32, 2608–2610 (2007). OPLEDP 0146-9592 10.1364/OL.32.002608 17767321

[53] Huisken Laboratory, FLAMINGO PROJECT: high-end microscopy inside and outside the optics lab.

[54] Nedbal J. et al., “Correction of time-resolved SPAD array measurements for accurate single-photon time-resolved biological imaging,” Proc. SPIE 11721, 117210T (2021). PSISDG 0277-786X 10.1117/12.2587755

[55] Schindelin J. et al., “Fiji: an open-source platform for biological-image analysis,” Nat. Methods 9, 676–682 (2012).1548-7091 10.1038/nmeth.2019 22743772 PMC3855844

[56] Randi E. B. et al., “Physiological concentrations of cyanide stimulate mitochondrial Complex IV and enhance cellular bioenergetics,” Proc. Natl. Acad. Sci. U. S. A. 118, e2026245118 (2021). PNASA6 0027-8424 10.1073/pnas.2026245118 33972444 PMC8157914

[r57] Shah A. T. et al., “Optical metabolic imaging of treatment response in human head and neck squamous cell carcinoma,” PLoS One 9, e90746–e90746 (2014). POLNCL 1932-6203 10.1371/journal.pone.0090746 24595244 PMC3942493

[58] Huang S. Heikal A. A. Webb W. W., “Two-photon fluorescence spectroscopy and microscopy of NAD(P)H and flavoprotein,” Biophys. J. 82, 2811–2825 (2002). BIOJAU 0006-3495 10.1016/S0006-3495(02)75621-X 11964266 PMC1302068

[59] White R. M. et al., “Transparent adult zebrafish as a tool for in vivo transplantation analysis,” Cell Stem Cell 2, 183–189 (2008). 10.1016/j.stem.2007.11.002 18371439 PMC2292119

[60] Yoo S. K. et al., “Differential regulation of protrusion and polarity by PI3K during neutrophil motility in live zebrafish,” Dev. Cell 18, 226–236 (2010).1534-5807 10.1016/j.devcel.2009.11.015 20159593 PMC2824622

[61] Houseright R. A. et al., “Myeloid-derived growth factor regulates neutrophil motility in interstitial tissue damage,” J. Cell Biol. 220, e202103054 (2021). JCLBA3 0021-9525 10.1083/jcb.202103054 34047769 PMC8167897

[62] Kristoffersen A. S. et al., “Testing fluorescence lifetime standards using two-photon excitation and time-domain instrumentation: rhodamine B, coumarin 6 and lucifer yellow,” J. Fluoresc. 24, 1015–1024 (2014). JOFLEN 1053-0509 10.1007/s10895-014-1368-1 24866152 PMC4070492

[r63] Freymüller C. et al., “Quenched coumarin derivatives as fluorescence lifetime phantoms for NADH and FAD,” J. Biophotonics 14, e202100024 (2021). 10.1002/jbio.202100024 33749988

[64] Sparks H. et al., “A flexible wide-field FLIM endoscope utilising blue excitation light for label-free contrast of tissue,” J. Biophotonics 8, 168–178 (2015). 10.1002/jbio.201300203 24573953 PMC4737404

[65] Sagar M. A. K. et al., “Optical fiber-based dispersion for spectral discrimination in fluorescence lifetime imaging systems,” J. Biomed. Opt. 25, 014506 (2019). JBOPFO 1083-3668 10.1117/1.JBO.25.1.014506 31833280 PMC6907392

[66] Sytsma J. et al., “Time-gated fluorescence lifetime imaging and microvolume spectroscopy using two-photon excitation,” J. Microsc. 191, 39–51 (1998). JMICAR 0022-2720 10.1046/j.1365-2818.1998.00351.x

[67] Heaster T. M. et al., “Autofluorescence imaging identifies tumor cell-cycle status on a single-cell level,” J. Biophotonics 11, e201600276 (2018). 10.1002/jbio.201600276 PMC5680147 28485124

[68] Bird D. K. et al., “Metabolic mapping of MCF10A human breast cells via multiphoton fluorescence lifetime imaging of the coenzyme NADH,” Cancer Res. 65, 8766–8773 (2005). CNREA8 0008-5472 10.1158/0008-5472.CAN-04-3922 16204046

[69] Barros-Becker F. et al., “Live imaging reveals distinct modes of neutrophil and macrophage migration within interstitial tissues,” J. Cell Sci. 130, 3801–3808 (2017). JNCSAI 0021-9533 10.1242/jcs.206128 28972134 PMC5702045

[70] “Canon develops SPAD sensor with world-highest 3.2-megapixel count, innovates with low-light imaging camera that realizes high color reproduction even in dark environments,” (2021).

[71] Pavia J. M. Wolf M. Charbon E., “Measurement and modeling of microlenses fabricated on single-photon avalanche diode arrays for fill factor recovery,” Opt. Express 22, 4202–4202 (2014). OPEXFF 1094-4087 10.1364/OE.22.004202 24663744

[72] Intermite G. et al., “Fill-factor improvement of Si CMOS single-photon avalanche diode detector arrays by integration of diffractive microlens arrays,” Opt. Express 23, 33777–33777 (2015). OPEXFF 1094-4087 10.1364/OE.23.033777 26832039

[73] Mai H. et al., “Development of a high-speed line-scanning FLIM microscope for biological imaging,” Opt. Lett. 48(8), 2042–2045 (2023). OPLEDP 0146-9592 10.1364/OL.482403 37058637

