# Supplementary material for: Light-sheet autofluorescence lifetime imaging with a single-photon avalanche diode array
Source: J Biomed Opt. 2023 Jun 21;28(6):066502. doi: 10.1117/1.JBO.28.6.066502 (PMC10284079; doi:10.1117/1.JBO.28.6.066502)
Supplement: Supplementary file 1 [file JBO_028_066502_SD001.pdf]

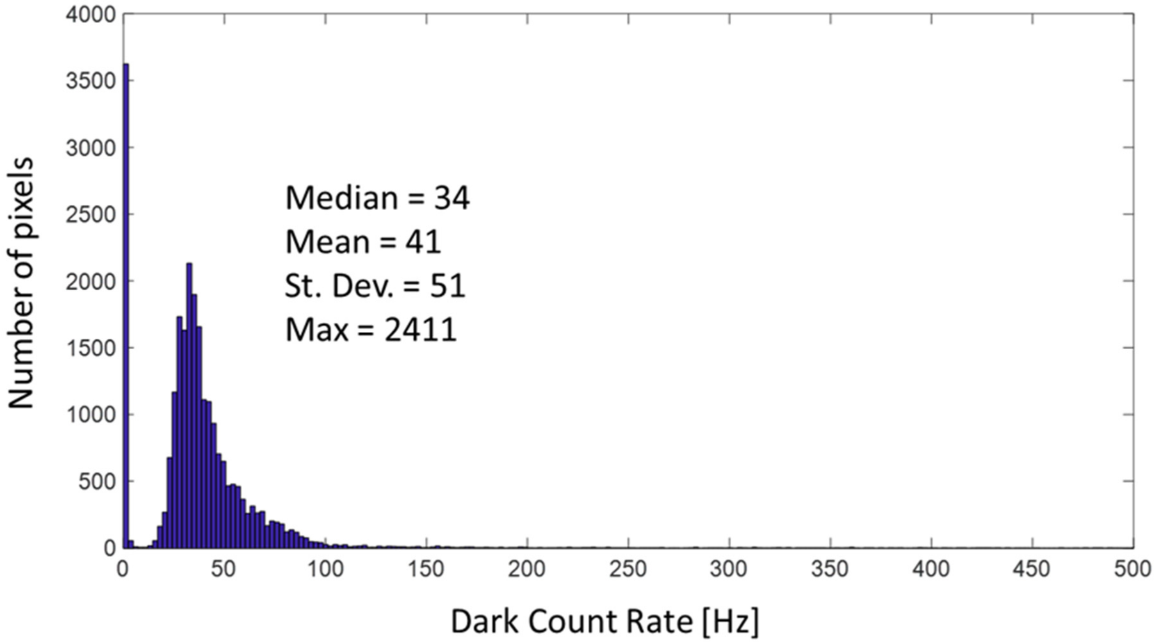

Supp. Fig. 1. Histogram of SPAD pixel dark count rates. The dark count rate of individual SPAD pixels was estimated from the non-zero tail of the pixel IRFs and the histogram of dark count rates across the sensor array was plotted. A median dark count rate of 34 counts per second (cps) is measured. Hot pixels with too high a DCR to produce useful data are called "screamers" and are turned off in the camera firmware. The screamers (not included in the above histogram) comprised 15% of all pixels on the FLIMera camera unit used in this work.

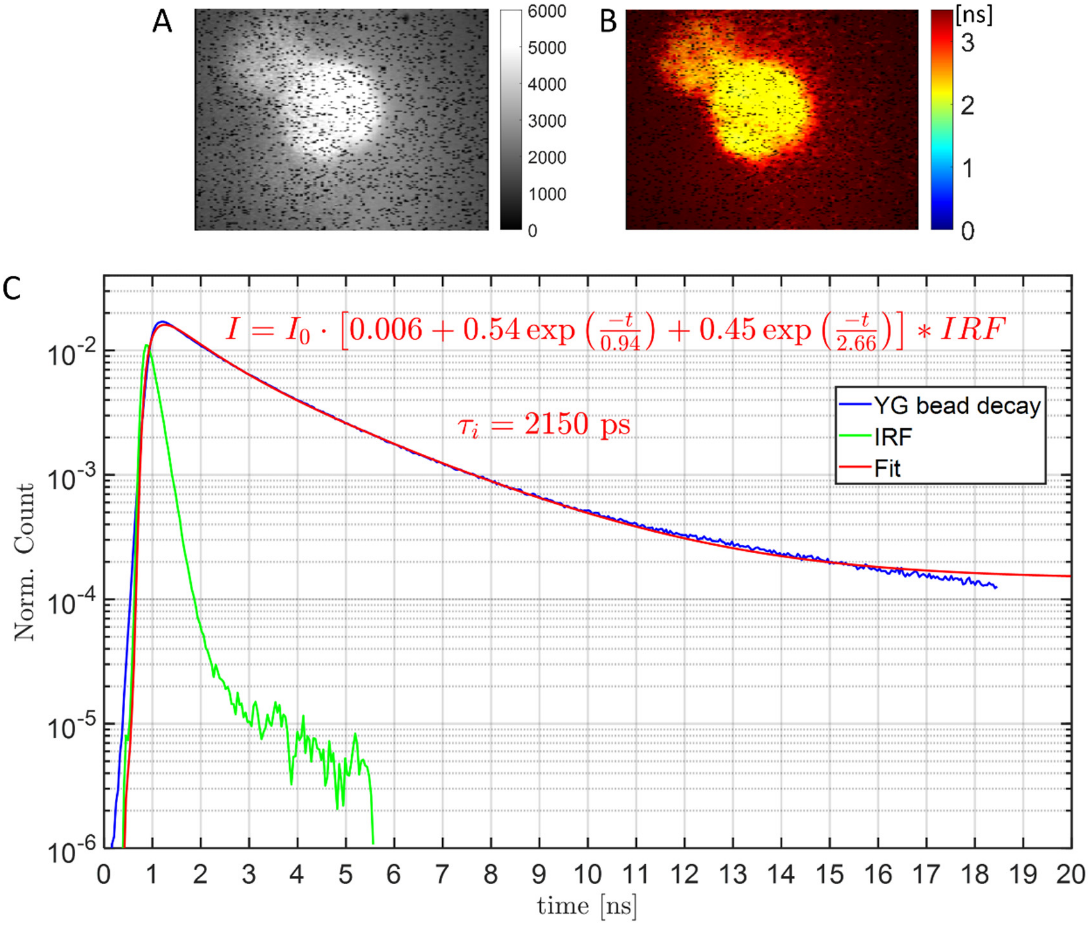

Supp. Fig. 2. Fluorescence lifetime imaging of Fluoresbrite® YG microspheres. A pair of 20 µm YG microspheres were imaged on the light sheet SPAD system with 0.4 mW excitation laser power and 1 second integration time using a 40×/1.13NA detection objective lens and a blue emission filter (440/80nm). The SPAD camera recorded 23 million photon counts per second from the beads. (A) shows the intensity image of the beads. (B) shows the intensity-weighted lifetime τ_i image of the beads which have values between 2.1-2.2 ns. Note the absence of pile-up effects despite the extremely high photon count rate from the bright YG beads. A laser-scanning microscope acquiring the same number of photons (23 million) in the same total integration time (1 sec) would suffer from pile-up effects that manifest as an over-sampling of the decay peak and an apparent reduced fluorescence lifetime artifact for the bright pixels. (C) shows the aggregate decay from all bead pixels and the biexponential model fit that yields an intensity-weighted mean fluorescence lifetime of 2.15 ns in agreement with values reported in the literature [65,66].

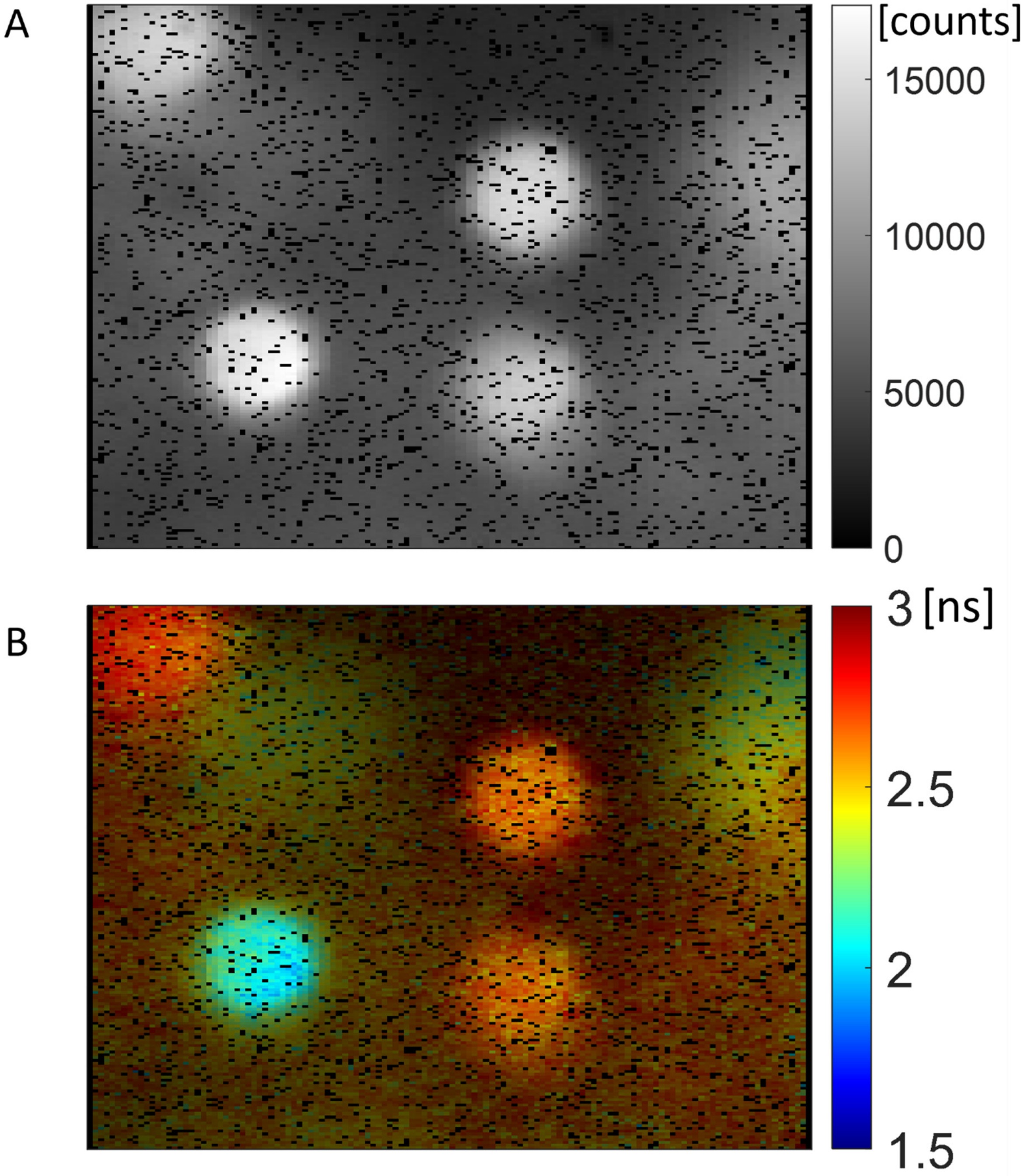

Supp. Fig. 3. Fluorescence lifetime imaging of Fluoresbrite® YG and BB microspheres. A mix of 10 µm YG and BB microspheres were imaged on the light sheet SPAD system with 0.2 mW excitation laser power and 3 second integration time using a 40×/1.13NA detection objective lens and a blue emission filter (440/80nm). The SPAD camera recorded 4-5 million photon counts from each microsphere. (A) shows the intensity image of the beads. (B) shows the intensity-weighted lifetime τ_i image of the beads which identifies the bead on the left as a YG microsphere with ~2.2 ns lifetime and the two beads on the right as BB microspheres with ~2.6 ns lifetime. The measured lifetime values agree with values reported in the literature [65,66].

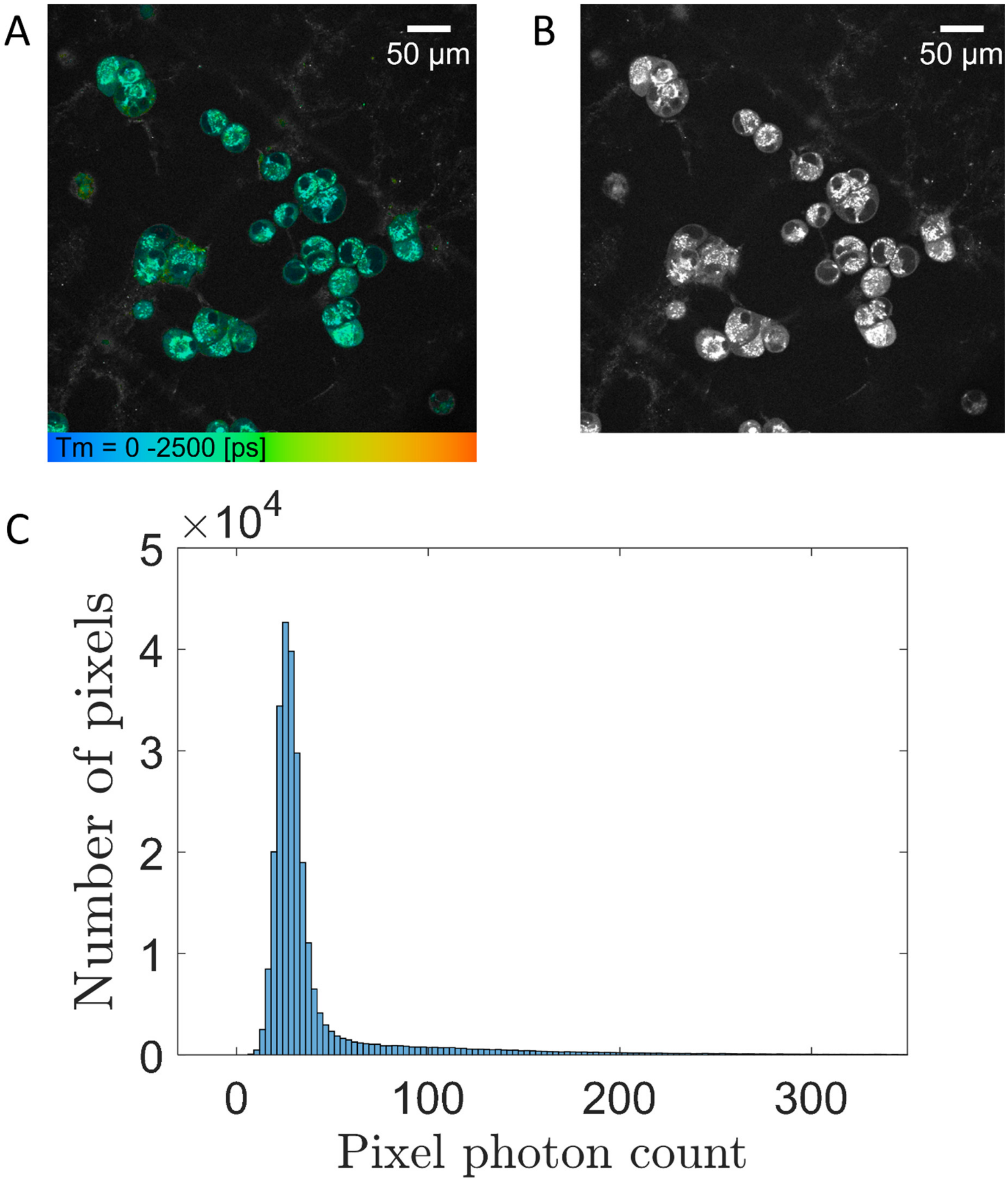

Supp. Fig. 4. Two-photon laser-scanning FLIM of PANC1 cells. (A) NAD(P)H mean fluorescence lifetime image of PANC1 cells with 512×512 pixels acquired on a two-photon microscope (Bruker Ultima 2P equipped with Becker & Hickl SPC-150 timing electronics) using typical imaging parameters [67] of 5 mW of 750 nm laser power and a Nikon 40× / 1.15NA WI objective lens over a 60 second integration time shows similar lifetime values to the light sheet SPAD array system. (B) shows the corresponding NAD(P)H intensity image. (C) shows the histogram of pixel photon counts. The top percentile pixel photon count in this image is 300 photons which corresponds to a photon count rate of ~1.3×10^6 cps. This value was used in Table 2 of the manuscript to calculate the required integration time for acquiring an image with the same number of photons and pixels as the light sheet FLIM system (which would be ~60 seconds or 6× longer than the light sheet system).
